# A Novel Inhaled Dry-Powder Formulation of Ribavirin Allows for Efficient Lung Delivery in Healthy Participants and Those with Chronic Obstructive Pulmonary Disease in a Phase 1 Study

**DOI:** 10.1128/AAC.02267-19

**Published:** 2020-04-21

**Authors:** Etienne F. Dumont, Amanda J. Oliver, Chris Ioannou, Julia Billiard, Jeremy Dennison, Frans van den Berg, Shuying Yang, Vijayalakshmi Chandrasekaran, Graeme C. Young, Anirban Lahiry, David C. Starbuck, Andrew W. Harrell, Alex Georgiou, Nathalie Hopchet, Andy Gillies, Stephen J. Baker

**Affiliations:** a GlaxoSmithKline, Collegeville, Pennsylvania, USA; b GlaxoSmithKline, Middlesex, United Kingdom; c Hammersmith Medicines Research Ltd., London, United Kingdom; d GlaxoSmithKline, Ware, United Kingdom; e GlaxoSmithKline, Bangalore, India; f Gillies Associates Limited, Harrogate, United Kingdom

**Keywords:** COPD, antiviral, exacerbations, novel inhalation powder, respiratory viruses, ribavirin

## Abstract

Chronic obstructive pulmonary disease (COPD) is an inflammatory lung condition, causing progressive decline in lung function leading to premature death. Acute exacerbations in COPD patients are predominantly associated with respiratory viruses. Ribavirin is a generic broad-spectrum antiviral agent that could be used for treatment of viral respiratory infections in COPD. Using the Particle Replication In Nonwetting Templates (PRINT) technology, which produces dry-powder particles of uniform shape and size, two new inhaled formulations of ribavirin (ribavirin-PRINT-CFI and ribavirin-PRINT-IP) were developed for efficient delivery to the lung and to minimize bystander exposure. Ribavirin-PRINT-CFI was well tolerated in healthy participants after single dosing and ribavirin-PRINT-IP was well tolerated in healthy and COPD participants after single and repeat dosing. Ribavirin-PRINT-CFI was replaced with ribavirin-PRINT-IP since the latter formulation was found to have improved physicochemical properties and it had a higher ratio of active drug to excipient per unit dose. Ribavirin concentrations were measured in lung epithelial lining fluid in both healthy and COPD participants and achieved target concentrations. Both formulations were rapidly absorbed with approximately dose proportional pharmacokinetics in plasma. Exposure to bystanders was negligible based on both the plasma and airborne ribavirin concentrations with the ribavirin-PRINT-IP formulation. Thus, ribavirin-PRINT-IP allowed for an efficient and convenient delivery of ribavirin to the lungs while minimizing systemic exposure. Further clinical investigations would be required to demonstrate ribavirin-PRINT-IP antiviral characteristics and impact on COPD viral-induced exacerbations. (The clinical trials discussed in this study have been registered at ClinicalTrials.gov under identifiers NCT03243760 and NCT03235726.)

## Abstract (precis)

Chronic obstructive pulmonary disease (COPD) is an inflammatory lung condition, causing progressive decline in lung function leading to premature death. Acute exacerbations in COPD patients are predominantly associated with respiratory viruses. Ribavirin is a generic broad-spectrum antiviral agent that could be used for treatment of viral respiratory infections in COPD. Using the Particle Replication In Nonwetting Templates (PRINT) technology, which produces dry-powder particles of uniform shape and size, two new inhaled formulations of ribavirin (ribavirin-PRINT-CFI and ribavirin-PRINT-IP) were developed for efficient delivery to the lung and to minimize bystander exposure.

## INTRODUCTION

Chronic obstructive pulmonary disease (COPD) is an inflammatory condition in the lung that causes a progressive decline in lung function, ultimately leading to a premature death. Primarily caused by smoking, COPD is typically diagnosed in patients over 40 years old and is currently the fourth leading cause of death in the United States (1). COPD patients experience acute exacerbations that increase in frequency as the disease progresses. Respiratory viruses are a major precipitant of these exacerbations, which cause significant morbidity and mortality in these patients (1, 2). Approximately 22 to 57% of acute exacerbations are associated with viruses such as human rhinovirus (HRV), respiratory syncytial virus (RSV), influenza virus (IFV), adenovirus (AdV), parainfluenza virus, human metapneumovirus, and picornavirus (1, 2). Early administration of antiviral agents at the onset of a respiratory viral infection may have the potential to reduce the severity or even prevent exacerbations in patients with COPD.

Ribavirin is a generic, broad spectrum nucleoside antiviral agent that is approved for the treatment of chronic hepatitis C virus by oral administration at a dose of 800 to 1,400 mg daily for up to 48 weeks. It is also approved for RSV bronchiolitis in pediatric patients by pulmonary inhalation as a nebulized formulation dosed 6 g daily administered over 2 or more hours for up to 7 days. Ribavirin is active against the key respiratory viruses that can cause acute exacerbations in COPD, including HRV, RSV, and IFV, with typical half-maximal effective concentrations (EC_50_) of 10 to 150 μM (unpublished data). Ribavirin is a prodrug which is actively transported into cells, where it is converted to its active metabolite ribavirin triphosphate (RTP) (3). The major limitations of the use of ribavirin are hemolytic anemia, due to the accumulation of RTP in red blood cells, and teratogenicity. This toxicity presents risks for current inhaled or nebulized formulations of ribavirin since the drug can disperse at sufficient concentrations into the immediate area closest to the patient and pose a risk of exposure to a pregnant bystander or to medical staff and caregivers when needed for patients who are unable to efficiently perform routine daily care. Exposure to nebulized ribavirin to health care personnel has been reported (4), in addition to reports of adverse events (AEs) in postmarketing surveillance (5).

We hypothesized that ribavirin could be useful in reducing or preventing virally induced acute exacerbations in COPD patients if administered at the onset of a respiratory viral infection. The approved formulations of ribavirin either will not achieve desired lung concentrations for efficacy (oral) or are not convenient to use due to the length of administration and risk of exposure to bystanders (nebulized). A dry-powder inhaled formulation would offer a convenient way to deliver ribavirin directly to the lungs, along with the opportunity to minimize toxicities in a manner that reduces the systemic exposure to the patient while not presenting a risk to bystanders. To achieve clinical efficacy, an antiviral state must be created in the lungs. Since HRV has the highest EC_90_ against ribavirin (150 μM), the minimum target was set to exceed this EC_90_ requiring a peak ribavirin concentration (*C*_max_) above 200 μM in the lung bronchiolar region. Although this peak concentration is transitory, it is anticipated that the intracellular concentration of the active form, RTP, would have a longer exposure and sufficient to impact viral replication. However, this is a high target concentration to achieve using a standard dry powder formulation; therefore, a different technology was explored.

Investigational ribavirin products were formulated using the Particle Replication In Nonwetting Templates (PRINT) technology by Liquidia Technologies, Inc. This approach produces dry-powder particles with a highly defined uniform shape and size. The PRINT formulation was anticipated to efficiently deliver a high concentration of ribavirin throughout the lungs, that would exceed the *C*_max_ in the bronchiolar region and establish an antiviral state. Two different formulations of ribavirin were produced. First, an amorphous formulation, called ribavirin-PRINT-CFI, was developed consisting of particles that are 1 μm in size and pollen shaped, containing 35% ribavirin with 55% trehalose and 10% trileucine as excipients (ratio 35:55:10 [wt/wt/wt]). These particles were loaded into capsules for use with a modified air inlet Rotahaler investigational inhalation device (a GlaxoSmithKline [GSK] proprietary device). A second improved formulation of ribavirin-inhalation powder, called ribavirin-PRINT-IP, was later developed, which is a crystalline formulation of cylinder (0.9 by 1 μm)-shaped particles containing 99% ribavirin and 1% polyvinyl alcohol (ratio 99:1 [wt/wt]). This formulation had a higher ratio of ribavirin to excipient and required less overall powder for inhalation to administer the same dose of ribavirin. This second formulation was used with a Monodose RS01 investigational inhalation device (Plastiape, Italy). Modeling was used to predict the dose of ribavirin needed to achieve peak concentrations above 200 μM in the bronchoalveolar fluid following 14 days of dosing by inhalation. The model estimated a daily dose of 60 mg of ribavirin (unpublished data).

Preclinical studies, which included bridging toxicology studies in rats and dogs by the inhalation route, were conducted to support progression of these formulations to the clinic (unpublished results). Here, we report the results of the phase I clinical studies with ribavirin-PRINT-CFI (amorphous form) and ribavirin-PRINT-IP (crystalline form). In the first study, single-escalating doses of ribavirin-PRINT-CFI were administered to healthy participants to evaluate the safety, tolerability, and lung and systemic pharmacokinetics (PK) of ribavirin-PRINT-CFI. In the second study, safety, tolerability, and lung and systemic PK of ribavirin-PRINT-IP were evaluated after single and repeated doses in healthy participants and those with moderate COPD.

The second study also evaluated the levels of ribavirin in room air and in the plasma of bystanders, since it was important to understand whether bystanders are exposed to teratogenic levels of ribavirin when this formulation is used by patients. Reducing bystander exposure to negligible levels would allow for the product to be conveniently and safely administered at home and not require a clinical setting.

## RESULTS

### Study 1: ribavirin-PRINT capsule for inhalation. (i) Study participant disposition and demographics.

The 60 enrolled participants (48 active, 12 placebo) were generally well matched across the treatment groups and all completed the study (Table 1).

**TABLE 1:** Participant demographics with amorphous ribavirin-PRINT-CFI

Demographics	Placebo (*n* = 12)	Amorphous ribavirin-PRINT-CFI						Overall (*n* = 60)
		7.5 mg (*n* = 6)	15 mg (*n* = 6)	30 mg (*n* = 6)	30 mg (*n* = 12)	60 mg (*n* = 6)	60 mg (*n* = 12)	
Mean age in yr (SD)	35.3 (11.41)	37.5 (13.46)	32.5 (9.61)	39.5 (12.28)	39.1 (12.80)	40.3 (12.29)	34.7 (14.91)	36.8 (12.31)
Sex, no. (%)								
Female	0	1 (17)	0	0	2 (17)	2 (33)	1 (8)	6 (10)
Male	12 (100)	5 (83)	6 (100)	6 (100)	10 (83)	4 (67)	11 (92)	54 (90)
Mean BMI, kg/m^2^ (SD)	24.33 (2.346)	27.07 (0.836)	25.08 (4.176)	26.33 (2.902)	25.91 (1.545)	24.43 (2.657)	24.62 (2.587)	25.26 (2.537)
Mean wt, kg (SD)	80.58 (10.411)	82.87 (7.545)	82.80 (15.558)	85.47 (13.844)	82.03 (9.721)	72.78 (13.298)	75.77 (8.649)	80.07 (11.084)
Race, no. (%)								
Asian	2 (17)	0	1 (17)	0	0	1 (17)	4 (33)	8 (13)
Black or African-American	2 (17)	2 (33)	0	2 (33)	2 (17)	0	1 (8)	9 (15)
White	8 (67)	3 (50)	4 (67)	4 (67)	9 (75)	5 (83)	6 (50)	39 (65)
Multiple	0	1 (17)	0	0	1 (8)	0	1 (8)	3 (5)
Not collected	0	0	1 (17)	0	0	0	0	1 (2)

### (ii) Pharmacokinetic results.

**FIG 1:**
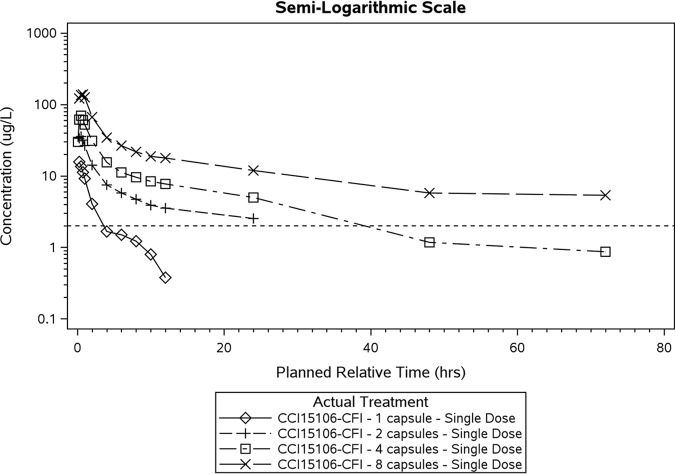
Mean Plasma Concentrations versus Time for Ribavirin-PRINT-CFI in Healthy Participants. Dashed line represents the lower limit of quantification of 2.0 ng/ml.

Following all doses (using a 7.5- to 60-mg single dose) of ribavirin-PRINT capsule for inhalation (ribavirin-PRINT-CFI), ribavirin was quantifiable in plasma within 15 min after inhalation, indicating a rapid absorption from lungs (Fig. 1 and Table 2). The maximal concentration (*C*_max_) increased with dose; however, due to the small sample size for each treatment, the lower limit of quantitation (LLQ) for the PK assessment, and the limited sampling times, the estimation of the AUC_t1–t2_ was reported instead of terminal half-life (*t*_1/2_) and AUC_inf._

**TABLE 2:** Summary of plasma ribavirin pharmacokinetic parameters in healthy participants with ribavirin-PRINT-CFI^*a*^

PK parameter (U)	Dose (mg)	*n*	Geometric mean	95% CI	%CVb
AUC_t1–t2_ (h⋅ng/ml)	7.5	6	9.73	5.30–17.9	63.3
	15	6	89.2	64.2–124	32.2
	30	6	268	161–448	51.9
	60	6	630	426–931	38.6
*C*_max_ (ng/ml)	7.5	6	13.6	7.29–25.5	65.4
	15	6	37.7	28.1–50.6	28.5
	30	6	65.0	41.4–102	45.1
	60	6	140	98.3–199	34.7
*T*_max_ (h)	7.5	6	0.258	0.25–0.75	
	15	6	0.417	0.25–0.75	
	30	6	0.500	0.25–0.62	
	60	6	0.750	0.42–1.00	

a The AUC_t1–t2_ parameter time interval by treatment is: 7.5 mg, AUC_0–1_; 15 mg, AUC_0–8_; 30 mg, AUC_0–24_; and 60 mg, AUC_0–24_. CI, confidence interval. %CVb = 100*sqrt [exp(SD**2) −1]. Data are presented to three significant figures. The *T*_max_ values are expressed as medians and ranges.

**FIG 2:**
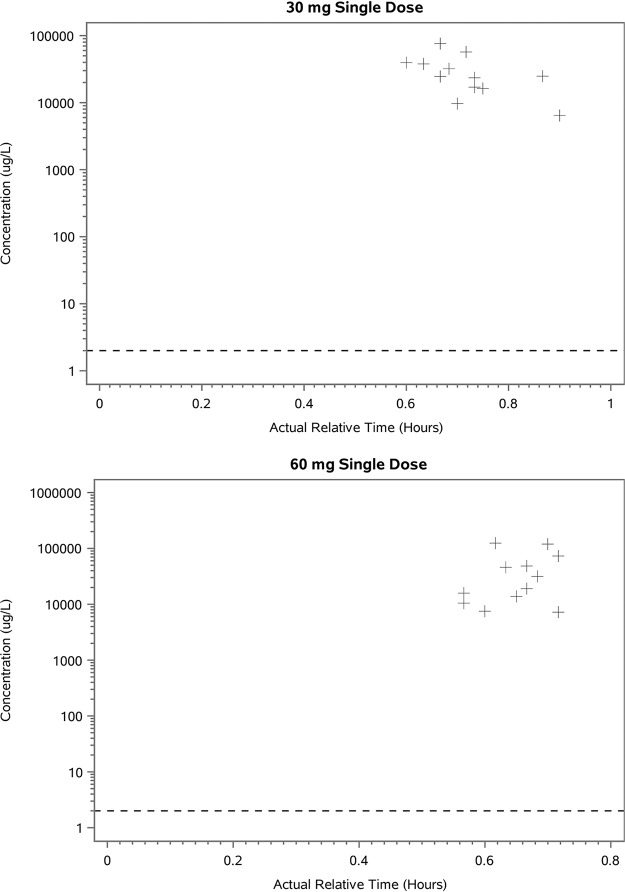
Lung ELF drug concentration versus time of dosing with ribavirin-PRINT-CFI. The lower limit of quantification (LLQ) is 2.0 μg/liter (2.0 ng/ml).

Lung epithelial lining fluid (ELF) drug concentrations measured between 45 and 60 min after dosing, with 30 mg (four capsules) or 60 mg (eight capsules) doses were medians of 101 and 104 μM, respectively, suggesting that the peak ELF concentration (*C*_max_) is likely to have exceeded the EC_50_ for HRV (36,600 ng/ml [150 μM]). The dose increases from 30 to 60 mg did not appear to result in a proportional increase in the lung ELF concentrations of ribavirin (Fig. 2 and Table 3).

**TABLE 3:** Summary of ribavirin lung ELF concentrations in healthy participants with ribavirin-PRINT-CFI^*a*^

Actual treatment (mg)	*N/n*	Visit	Planned BAL time point (h)	Concn, μg/liter (μM)^*b*^			
				Mean	SD	Median	Range
30	12/12	Part 1 day 1	0.25–1	30,400 (125)	20,000 (82.0)	24,800 (101)	645–76,000 (26.5–311)
60	12/12	Part 1 day 1	0.25–1	43,200 (177)	42,100 (172)	25,400 (104)	7,260–125,000 (29.8–511)

a Lung ELF drug concentration is the concentration once the plasma urea (pre-BAL)/BAL urea dilution factor is applied. SD, standard deviation; ELF, epithelial lining fluid; *N*, total number of participants; *n*, number of participants with observations. Data are presented to three significant figures.

b 1 μM = 244 μg/liter.

### (iii) Safety.

Overall, the frequency and types of AEs were similar across all doses of Ribavirin-PRINT-CFI and placebo (Table 4). The majority of AEs were reported by single participants in all treatment groups, including placebo. Headache (*n* = 11) was the most common AE throughout the study with a similar frequency across all groups; 9 cases were considered drug-related by the investigator. Mild cough was reported in 3 participants in the ribavirin treatment groups only. All AEs were mild to moderate in intensity. There were no withdrawals due to AEs, deaths or serious AEs. There were no clinically significant changes in clinical laboratory tests, spirometry, vital signs or electrocardiograms (ECGs).

**TABLE 4:** Summary of adverse events reported by ≥2 participants dosed with amorphous ribavirin-PRINT-CFI

Preferred term	No. (%)							
	Placebo (*n* = 12)	Amorphous ribavirin-PRINT-CFI cohorts						Overall (*n* = 60)
		7.5 mg (*n* = 6)	15 mg (*n* = 6)	30 mg (*n* = 6)	30 mg (*n* = 12)	60 mg (*n* = 6)	60 mg (*n* = 12)	
Any event	7 (58)	3 (50)	3 (50)	2 (33)	4 (33)	1 (17)	5 (42)	25 (42)
Any drug-related AE	2 (17)	1 (17)	2 (33)	1 (17)	2 (17)	1 (17)	3 (25)	12 (20)
Headache	2 (17)	1 (17)	2 (33)	0	3 (25)	0	3 (25)	11 (18)
Cough	0	1 (17)	1 (17)	0	0	1 (17)	0	3 (5)
Toothache	1 (8)	0	0	1 (17)	0	0	0	2 (3)
Influenza-like illness	1 (8)	0	0	0	0	0	1 (8)	2 (3)
Pyrexia	1 (8)	0	0	0	1 (8)	0	0	2 (3)
Musculoskeletal chest pain	0	0	0	0	0	0	2 (17)	2 (3)

### Study 2: ribavirin-PRINT inhalation powder.

**FIG 3:**
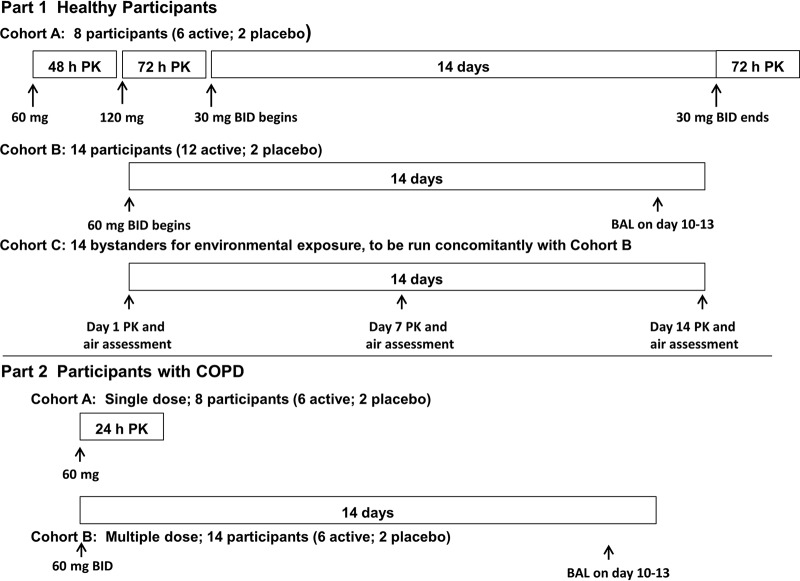
Study design with crystalline ribavirin-PRINT-IP (cohort B, multiple dose; 8 participants [6 active; 2 placebo]).

The study design is shown in Fig. 3.

**(i) Participant disposition and demographics.** The healthy participants in part 1 were well matched across the three cohorts, and all completed the study as planned. In part 2, the COPD patients were also well matched in the two dosing cohorts. The demographics are summarized in Table 5 and Table 6.

**TABLE 5:** Participant demographics with crystalline ribavirin-PRINT-IP: part 1, healthy participants

Demographic	Dosing cohorts		Bystanders
	Cohort A^*a*^ (*n* = 8)	Cohort B^*b*^ (*n* = 14)	Cohort C (*n* = 14)
Mean age, yr (SD)	41.1 (9.49)	36.1 (10.97)	37.4 (12.9)
Sex, no. (%)			
Female	0	0	1 (7)
Male	8 (100)	14 (100)	13 (93)
Mean (SD)			
BMI^*c*^ (kg/m^2^)	25.34 (2.8)	26.19 (2.7)	24.12 (2.7)
Ht (cm)	180.3 (6.0)	177.0 (7.3)	177.6 (7.3)
Wt (kg)	82.45 (11.7)	82.40 (11.9)	76.25 (11.4)
Ethnicity, no. (%)			
Hispanic or Latino	0	1 (7)	0
Not Hispanic or Latino	8 (100)	13 (93)	14 (100)
Race, no. (%)			
Black or African-American	1 (13)	2 (14)	3 (21)
White: white/Caucasian/European heritage	7 (88)	11 (79)	9 (64)
Multiple	0	1 (7)	2 (14)

a Ratio of active:placebo in cohort A = 3:1.

b Ratio of active:placebo in cohort B = 6:1.

c BMI, body mass index.

**TABLE 6:** Participant demographics with crystalline ribavirin-PRINT-IP: part 2, COPD patients

Demographic	Cohort A, single dose (*n* = 8)^*a*^	Cohort B, multiple dose (*n* = 8)^*b*^
Mean age, yr (SD)	69.9 (6.27)	65.0 (5.13)
Sex, no. (%)		
Female	2 (25)	2 (25)
Male	6 (75)	6 (75)
Mean (SD)		
BMI^*c*^ (kg/m^2^)	23.78 (4.9)	24.43 (2.5)
Ht (cm)	171.4 (9.2)	169.1 (7.5)
Wt (kg)	70.55 (18.7)	69.95 (9.0)
Ethnicity, no. (%)		
Hispanic or Latino	1 (13)	0
Not Hispanic or Latino	7 (88)	8 (100)
Race, no. (%)		
White: white/Caucasian/European heritage	8 (100)	8 (100)

a Ratio of active to placebo in cohort A = 6:2.

b Ratio of active to placebo in cohort B = 12:2.

*^c^*BMI, body mass index.

**FIG 4:**
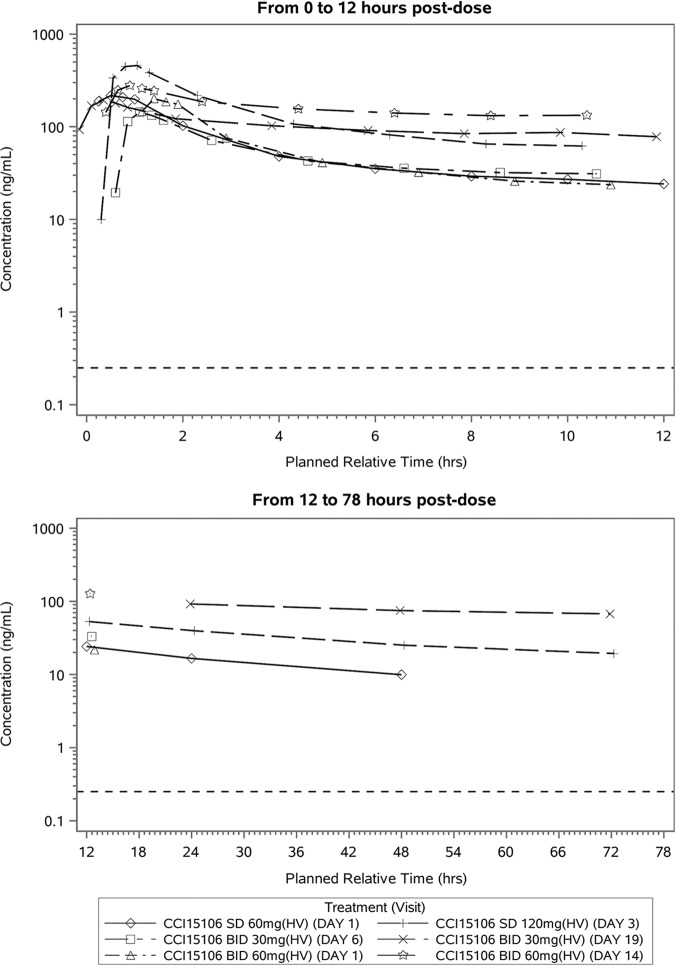
Mean plasma concentrations versus time with ribavirin-PRINT-IP in healthy participants. The dashed line represents the lower limit of quantification of 0.25 ng/ml.

**(ii) Pharmacokinetic results—plasma concentrations.** Ribavirin was quantifiable in plasma of all healthy and COPD patients who were deliberately administered ribavirin-PRINT inhalation powder (ribavirin-PRINT-IP) at all administered doses at all time points. In healthy participants, following single and repeated inhaled doses of ribavirin-PRINT-IP at all doses, maximum ribavirin plasma concentrations were reached ∼0.5 h after dosing (Table 7 and Fig. 4). The between-participant variability was low to moderate after both single and repeat dosing. In healthy participants, systemic exposure after single dosing, based on *C*_max_ and AUC estimate, increased in an approximately dose proportional manner. The AUC from 0 to 12 h (AUC_0–12_) was shown to increase approximately dose proportionally between 60 and 120 mg, while a slightly less than dose-proportional increase was observed compared to 30 mg. This may be due to the insufficient washout before starting 30 mg twice daily (BID) in part 1 of cohort A.

**TABLE 7:** Summary of derived plasma ribavirin pharmacokinetic parameters in healthy participants with crystalline ribavirin-PRINT-IP^*a*^

PK parameter (U)	Dose frequency, size (mg)	Visit (day)	*N/n*	Geometric mean	95% CI	%CVb
Cohort A						
AUC_0−12_ (h⋅ng/ml)	SD, 60	1	6/6	715	594–861	17.8
	SD, 120	3	6/6	1,490	1,140–1,950	25.8
	BID, 30	6	6/6	578	464–721	21.3
*C*_max_ (ng/ml)	SD, 60	1	6/6	232	188–287	20.2
	SD, 120	3	6/6	508	382–674	27.6
	BID, 30	6	6/6	143	113–182	23.2
		19	6/6	194	166–227	15.1
*T*_last_ (h)	SD, 60	1	6/6	47.8		
	SD, 120	3	6/6	71.8		
	BID, 30	6	6/6	11.9		
		19	6/6	11.9		
*T*_max_ (h)	SD, 60	1	6/6	0.500	0.25–1.00	
	SD, 120	3	6/6	0.625	0.25–0.75	
	BID, 30	6	6/6	0.500	0.50–0.75	
		19	6/6	0.500	0.25–0.50	
						
Cohort B						
AUC_0–τ_ (h⋅ng/ml)	BID, 60	1	12/12	565	418–763	50.2
		14	12/8	2,060	1,820–2,340	15.2
*C*_max_ (ng/ml)	BID, 60	1	12/12	189	130–273	63.5
		14	12/11	285	221, 367	39.0
*T*_last_ (h)	BID, 60	1	12/12	11.7	11.7–11.8	
		14	12/11	11.7	11.7−12.0	
*T*_max_ (h)	BID, 60	1	12	0.633	0.50–1.00	
		14	11	0.500	0.30–1.00	

a *T*_max_ and *T*_last_ are expressed as medians and ranges, where appropriate. The *T*_last_ ranges in cohort A are not presented since they were the same for all subjects at all times. Tau (τ) = 12 h, the dosing interval for the BID dosing regimen. SD, single dose, BD, twice daily. *N*, total number of participants; *n*, number of participants with observations.

Mean plasma ribavirin concentrations continued to increase by day 14, indicating that a steady-state plasma concentration was not reached after 14 days of repeat BID administration of 30- and 60-mg doses to healthy participants and BID administration of 60 mg to COPD patients. Based on the AUC_0–τ_ (τ = 12 h) ratios for day 14/day 1, the observed accumulation was 4.07 and 4.53 after BID administration of 60 mg BID in healthy participants and in COPD patients, respectively. For cohort A, the accumulation ratio based on the AUC_0–τ_ for day 19/day 6 (the last and first dosing days, respectively) was 2.10 following administration of 30 mg BID in healthy participants. Each of the 90% confidence intervals for the accumulation ratio excluded 1, resulting in a statistically significant accumulation ratio at the 10% significance level. The *t*_1/2_ of plasma ribavirin was considered unreliable due to limited sampling period.

After dosing with 60 mg BID for 14 days, COPD patients had approximately 1.5-fold higher systemic concentrations of ribavirin versus the healthy volunteer group (Table 8). The shape of the PK profiles for all treatment groups in healthy participants and patients with COPD appeared to be similar.

**TABLE 8:** Comparison of healthy participants and COPD patient concentration of RIBAVIRIN in plasma^*a*^

Treatment group, dose	PK parameter (units)	*N*	*n*	Visit (day)	Geometric mean	95% CI	%CVb
BID, 60 mg (HV)	*C*_max_ (ng/ml)	12	12	1	189	130–273	63.5
			11	14	285	221–367	39.0
	AUC_0–τ_ (h.ng/ml)	12	12	1	565	418–763	50.2
			8	14	2,060	(1,815, 2,337)	15.2
BID, 60 mg (COPD)	*C*_max_ (ng/ml)	8	8	1	178	119–267	51.4
			8	14	423	326–549	31.9
	AUC_0–τ_ (h.ng/ml)	8	8	1	687	499–947	39.8
			7	14	3,087	(2,393, 3,982)	28.1

a *N*, total number of participants; *n*, number of participants with observations.

**(iii) Pharmacokinetic results—bystander participants and bystander exposures.** Ribavirin plasma concentrations for all bystanders were all below the assay LLQ of 0.25 ng/ml. Ribavirin concentrations in the air within the bystander’s breathing zone, with bystanders positioned close to the dosing participants, ranged from below detection limit to 9.22 μg/m^3^. The majority of these samples (88%) had concentrations of <5 μg/m^3^. More than 30% of the samples were below the detection limit. Calculated 8-h average exposures (assuming negligible exposure when leaving the procedure room) were all <1 μg/m^3^, which is 10% of the GSK occupational exposure limit of 10 μg/m^3^ calculated based on internal and external guidance (6–10).

**FIG 5:**
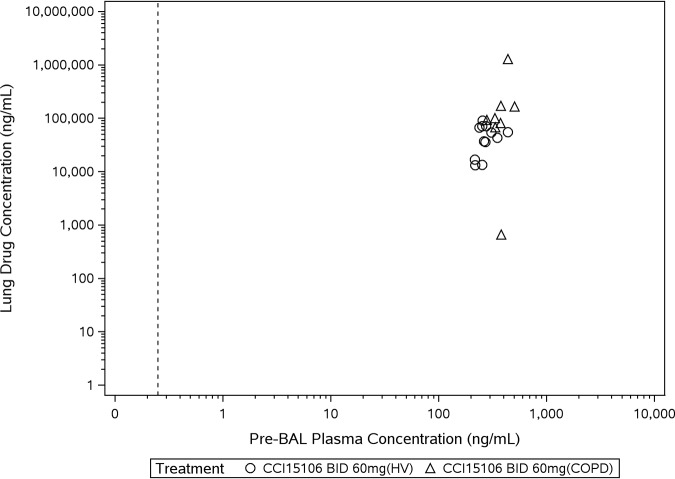
Scatter plot of ELF drug concentration versus plasma drug concentration with ribavirin-PRINT-IP. The dashed line represents the lower limit of quantification of 0.25 ng/ml.

**(iv) Pharmacokinetic results—lung epithelial lining fluid concentrations.** Lung ELF drug concentrations were estimated from measured bronchoalveolar lavage (BAL) fluid samples taken within up to 60 min after dosing with 60 mg BID, on one single day between days 10 and 13 in both healthy and COPD participants (Fig. 5). Pre-BAL ribavirin plasma concentrations were greater in COPD patients (part 2) compared to healthy participants (part 1). Lung ELF ribavirin concentrations were appreciably greater than ribavirin plasma concentrations (Tables 8 and 9).

**TABLE 9:** Summary of lung ELF ribavirin concentrations with crystalline ribavirin-PRINT-IP^*a*^

Participants	*N/n*	Mean (SD), μg/liter	Median, μg/liter	Range, μg/liter
Healthy, part 1	12/12	47,300 (25,200)	48,560	13,300–91,200
COPD, part 2	8/8	247,000 (427,000)	96,700	662–1,300,000

a For part 1 (healthy participants) and part 2 (COPD patients), BAL samples were collected once between days 10 and 13 by 1 h after the first dose of the day. The lung drug concentration is the concentration once the plasma urea (pre-BAL)/BAL urea dilution factor is applied. *N*, total number of participants; *n*, number of participants with observations.

**(v) Safety.** Overall, a small number of healthy participants reported AEs (Table 10). There were no reported AEs after single doses of 60 and 120 mg. The majority of AEs were reported by single participants across all doses, including the placebo. The greatest number of AEs (*n* = 8, 67%) was reported in the 60 mg BID repeat dosing cohort. Of the total 14 AEs, 5 AEs were considered possibly treatment related. Two severe AEs (syncope and vomiting) in one healthy participant were related to the BAL procedure, most likely due to the invasive nature of this procedure and the preceding sedation. There were no withdrawals due to the study medication. No clinically meaningful changes in clinical laboratory tests, vital signs, spirometry, or ECGs were observed. Spirometry measurements were within normal range for healthy participants and lower for participants with COPD, as expected. There were no serious AEs (SAEs) reported, and no AEs led to withdrawal from the study.

**TABLE 10:** Summary of adverse events reported by ≥2 healthy participants dosed with crystalline ribavirin-PRINT-IP

Preferred term	*n* (%)^*a*^				
	Placebo (*N* = 4)	60 mg, SD (*N* = 6)	120 mg, SD (*N* = 6)	30 mg, BID (*N* = 6)	60 mg, BID (*N* = 12)
Any AE	4 (100)	0	0	2 (33)	8 (67)
Headache^*b*^	0	0	0	0	3 (25)
Malaise	0	0	0	0	3 (25)
Feeling hot	0	0	0	0	2 (17)
Vomiting	0	0	0	0	2 (17)

a *N*, total number of participants; *n*, number of participants with observations.

b Considered related to treatment by the investigator.

In COPD patients, AEs were only reported in participants who received ribavirin-PRINT-IP at 60 mg BID. Eight participants reported a total of seven single AEs (88%) and included the following: dyspnea, exertional dyspnea, oropharyngeal pain, wheezing, plantar fasciitis, headache, presyncope, chest discomfort, constipation, gastroesophageal reflux disease, ear pain, dry skin, and macular rash. All AEs were mild to moderate in severity, and none were considered by the investigator to be related to treatment. There was no evidence of bronchospasm or cough.

## DISCUSSION

Aerosolized ribavirin for inhalation solution has been available since 1985 for the treatment of pediatric RSV bronchiolitis (5). Oral formulations of ribavirin have also been approved for treatment of chronic hepatitis C viral infections in adults (11, 12). COPD patients with acute exacerbation caused by viral infections could benefit from early administration of ribavirin due to its broad-spectrum antiviral properties to prevent or minimize virally associated exacerbations. Since the standard nebulized product is not convenient for drug delivery to the lungs of COPD patients and poses a teratogenic risk to bystanders, a new delivery system of ribavirin was explored that would allow for convenient administration of ribavirin in a home setting. Thus, using the PRINT technology, two novel highly dispersible dry-powder inhaled formulations of ribavirin were developed and studied in healthy participants and those with COPD. The PRINT technology produces dry-powder particles of uniform shape and size for delivery to the site of action. The first formulation, ribavirin-PRINT-CFI, contained 35% ribavirin and 65% excipients. In contrast, the second formulation, ribavirin-PRINT-IP, was more stable and consisted of 99% ribavirin, thus allowing for a decrease in total inhaled powder.

Ribavirin-PRINT-CFI was well tolerated in healthy participants after single dosing, and ribavirin-PRINT-IP was well tolerated in healthy and COPD participants after single and repeat dosing. None of the reported AEs were severe, serious, or led to withdrawals. There were no other clinically significant findings with these new ribavirin-PRINT formulations dosed for up to 14 days to mimic expected duration of treatment. Most importantly, there were no reports of bronchospasm with either of the formulations at any dose, which would be a potential concern for administration of aerosolized formulations to COPD patients. The majority of the clinical experience was obtained with the optimized ribavirin-PRINT-IP formulation.

Ribavirin concentrations in ELF indicated that the PRINT particles effectively delivered inhaled ribavirin to the lung. Lung ELF ribavirin concentrations were measurable in both healthy and COPD participants. Since efficacy was not an objective of this study, further studies would need to be conducted to determine whether the ribavirin concentrations in the lung are sufficient for antiviral activity, although the maximum concentrations achieved in lung ELF with the ribavirin-PRINT-IP formulation were estimated to be above the EC_50_ values for viruses commonly involved in exacerbations.

The pharmacokinetic profile indicated that ribavirin rapidly reached systemic circulations following single and repeat doses of ribavirin-PRINT-IP and was dose proportional in healthy participants. However, overall, comparing to the existing oral/nebulized dosing of ribavirin, the systemic ribavirin concentrations were low following ribavirin-PRINT-CFI administration, thus minimizing the risk and likelihood of the occurrence of hemolytic anemia, a known side effect with chronic ribavirin administration (5, 11, 12).

It is well established that ribavirin is teratogenic with black box warnings and contraindications in pregnant women being described in ribavirin labels and product information leaflets (5, 11, 12). Therefore, a major concern for inhaled ribavirin was bystander exposure especially to pregnant women, potentially leading to human fetal abnormalities. Rat and rabbit embryo-fetal development studies, described in the FDA Summary Basis of Approval for Rebetol (13), can be used to predict the ribavirin plasma concentrations that are unlikely to be associated with a teratogenic risk. In rats, the most sensitive reproductive toxicology species, at ribavirin doses ≥1 mg/kg, fetal abnormalities were evident as gross visceral and skeletal abnormalities with late fetal deaths twice those on control. The corresponding ribavirin plasma *C*_max_ at the rat no-effect level of 0.3 mg/kg was reported as 0.06 μM or 14.6 ng/ml. All human bystander plasma concentrations measured in this study were not quantifiable in the human plasma assay with the lower limit of quantification of 0.25 ng/ml, which is >58-fold lower than the ribavirin *C*_max_ concentration in the most sensitive species used to test embryo-fetal toxicity. There is no specific guidance that can be used to advise a safe bystander exposure. FDA guidance on Reproductive and Development toxicities (14), however, advises an increased concern for human reproductive or developmental toxicity when exposure ratios are <10-fold and decreased concern when the ratios are >25-fold. The ratio of >50-fold reported here is consistent with concentrations that would be considered to be of low or negligible concern.

In addition to measuring bystander plasma ribavirin concentrations, airborne ribavirin concentrations were measured in the bystander’s breathing zone to assess potential exposure arising from airborne drug release during handling and disposal of the capsules. A sampling plan was agreed where 15-min samples were collected on days 1, 7, and 14 of a dosing cohort to monitor temporal differences, which included 42 personal exposure samples taken during four separate dosing groups (plus 84 static samples). Of these, 30% of personal exposure samples were below detection limits with a maximum measured concentration of 9.22 μg/m^3^. The majority (88%) of personal exposure samples contained ribavirin concentrations of <5 μg/m^3^. When the results were time-weighted over 8 h to compare with the internal GSK exposure limit of 10 μg/m^3^, all results were <10% of the limit, indicating a low or insignificant risk of exposure via inhalation. Considering both the systemic exposure and the environmental air monitoring data, it was concluded that inhalation administration of ribavirin-PRINT-IP in nonclinical settings would be deemed acceptable and constituted a negligible risk to bystanders.

In conclusion, the uniform shape and size of the dry-powder particles in the PRINT formulations allow for an efficient and convenient delivery of ribavirin to the lungs while minimizing systemic exposure. Following dosing of participants, unintended exposure of ribavirin to bystanders was found to be negligible based on both the plasma and the airborne ribavirin concentrations, providing a convenient formulation for patients to use at home and following an onset of upper respiratory symptoms suggestive of a viral infection. In addition, due to the higher ratio of active drug to excipient per unit dose, only two capsules of the ribavirin-PRINT-IP formulation were required to deliver a dose of the drug that met the target concentration in the lung. Further development is required to explore the efficacy of ribavirin-PRINT-IP.

## MATERIALS AND METHODS

The studies described here were conducted per the ethical principles of “good clinical practice” and the Declaration of Helsinki after obtaining a written informed consent from each subject. The protocols and the informed consents were approved by an independent ethics committee.

### Study population.

Both studies enrolled healthy participants, including males or females of nonreproductive potential, between 18 and 65 years of age (inclusive), with body weights of ≥50 kg for males and ≥45 kg for females. In study 1, the body mass index (BMI) was between 19 and 31 kg/m^2^ for males and females, while in study 2, the BMI was specified at 17 to 31 kg/m^2^ for females.

Study 2 also included participants with COPD in Part 2. The eligibility criteria included a diagnosis of moderate COPD (GOLD class II). Participants had spirometry at screening and were eligible if their postbronchodilator forced expiratory volume in 1 second (FEV_1_) was ≥50% and <80% predicted normal, and postbronchodilator FEV_1_/forced vital capacity (FVC) was <0.7 (1, 15). These COPD participants were smokers (or ex-smokers) with a smoking history of at least 10 pack years. Participants were excluded if they had poorly controlled COPD, a respiratory tract infection requiring antibiotic treatment in the 4 weeks prior to screening, or a diagnosis of active tuberculosis, lung cancer, or other interfering respiratory disorder. The use of short- and long-acting inhaled bronchodilators was allowed, but participants were required to discontinue their medications for periods pre- and postdose and prior to spirometry procedures.

The use of didanosine or azathioprine was an exclusion criterion for all participants in both studies.

### Study design. (i) Design for study 1—ribavirin-PRINT capsule for inhalation.

Study 1 was originally designed as a sequential three-part, double-blind (sponsor unblind), randomized, placebo-controlled, single and repeated escalating dose study with ribavirin-PRINT capsule for inhalation (ribavirin-PRINT-CFI) (NCT03243760). Part 1 was a single-dose escalation study in healthy participants. In parts 2 and 3, which were to be conducted sequentially, ribavirin-PRINT-CFI was planned to be administered as repeated doses in healthy participants and as single doses in participants with moderate COPD. However, due to benefits offered by a concurrently developed and improved second formulation, ribavirin-PRINT-IP, parts 2 and 3 were not carried out. All participants in this study were dosed in a negative pressure enclosure to minimize unintended exposure of the study personnel to ribavirin.

Participants were randomized into 6 cohorts to receive either ribavirin-PRINT-CFI or matching PRINT particles without the active ingredient (placebo), as shown in Table 11. In cohorts A, B, C, and E, 8 healthy participants were randomized per cohort to receive active or placebo in a 3:1 ratio. In cohorts D and F, 14 participants were randomized to receive active or placebo in a 6:1 ratio. In cohorts D and F, a BAL was performed to evaluate levels of ribavirin in the epithelial lining fluid (ELF) and epithelial cell pellets. A modified air inlet ROTAHALER investigational inhalation device was used for administration of the study drug in this study (GSK proprietary device).

**TABLE 11:** Dosing schedule with amorphous ribavirin-PRINT-CFI

Cohort^*a*^	Dose in mg (no. of capsules)	No. of participants (active/placebo)
A	7.5 (1)	6/2
B	15 (2)	6/2
C	30 (4)	6/2
D^*a*^	30 (4)	12/2
E	60 (8)	6/2
F^*a*^	60 (8)	12/2

a Cohorts D and F, which involved a BAL procedure, were dosed after assessing the tolerability of the 30- and 60-mg dose, respectively, in cohorts C and E.

In cohort A only, the first three participants received the first dose of 7.5 mg or placebo (one capsule) on one day, followed by the remaining five participants at least one day later. Participants were discharged the next day and returned for a follow-up visit approximately 30 days after dosing. The doses were escalated in cohort A to cohort D from 7.5 to 30 mg, as shown in Table 11. Dose escalation between each cohort was based on a review of safety, tolerability, and PK data after each cohort. The study sponsor was unblinded at the aggregate level for all decision making during the study. After completion of dosing in cohort D (30 mg), a comprehensive review of all data from prior cohorts was conducted before dose escalation to 60 mg in cohorts E and F. A BAL was performed for cohorts D (30 mg) and F (60 mg) as soon as possible after dosing (within 1 h) to be close to the anticipated *T*_max_ for lung exposure. In part 1, serial PK samples were collected up to 24 hours after single dosing.

### (ii) Design for study 2—ribavirin-PRINT inhalation powder.

Study 2 was a two-part, double-blind (sponsor unblind), randomized, placebo-controlled, single and repeated escalating dose study (NCT03235726). Figure 3 summarizes the study design.

Part 1 investigated single and repeat ascending doses of ribavirin-PRINT inhalation powder (ribavirin-PRINT-IP) in healthy participants and also environmental contamination and bystander exposure. In part 1, cohort A, 8 healthy participants were randomized to receive active or placebo in a 3:1 ratio. In cohort B, 14 healthy participants were randomized to receive active or placebo in a 6:1 ratio, and in cohort C, 14 healthy participants were enrolled as bystanders for environmental exposure, run concomitantly with cohort B. The doses for part 1 were based on the favorable safety profile from the first study described above as well as preclinical safety data (unpublished results). Participants in cohort A were exposed to single doses of 60 and 120 mg, followed by 30 mg BID for 14 days. Dosing occurred in a negative pressure enclosure. In cohort B, participants received 60 mg BID for 14 consecutive days. At each dose, two bystander participants (cohort C) were seated in close proximity to two dosing participants from cohort B in one room. Bystanders were positioned in different orientations to dosing participants; some sat opposite facing each other (ca. 1 m, across table) and some were side-by-side (50 cm, same side of table). Bystanders handled capsules, loaded the capsules into the device, and handed it to the dosing participant; after dosing, the bystander opened the device to check that the capsule had been punctured and observed if any residual drug powder was present. Empty capsules were left in open tray on the table for the rest of the exposure monitoring period (15 min). Participants in cohort B were dosed without the use of a negative pressure enclosure to allow estimation of bystander exposure to ribavirin. Before participants were dosed without the enclosure, all study personnel left the room and did not reenter until 15 min after dosing. Participants were observed through a window in the door for the duration of sampling to note relevant contextual information to assist in the interpretation of the exposure measurements. Serial PK samples were collected up to 12 h on days 1, 3, 6, and 19. On days 4 and 5, one sample was collected in the morning. On days 2, 9, 11, 13, 15, and 17, PK samples were collected once before the morning dose (at trough) to assess steady-state following repeated dosing.

In cohort B, static air samples were collected on filters within air pumps positioned in two locations in the room during and after the first daily doses on days 1, 7 and 14. Filters were held in Institute of Occupational Medicine (IOM) sampling heads attached to sampling pumps by tubing; the heads were positioned in the participant’s breathing zone for measurement of ribavirin concentrations. Samples were collected over 20 and 60 min, following dosing in each location. An additional set of samples were collected over 60 min, 1 day before the start of dosing on day 1 to provide a background benchmark for reference. On days when static air sampling was performed, the dosing sessions were separated by the time necessary to collect the 60-min air sample. Sampling devices were used to collect samples for the measurement of ribavirin concentrations. Air sample analysis was performed by the Bureau Veritas laboratory (Bureau Veritas North America, Lake Zurich, IL), an industrial hygiene analysis laboratory accredited by the American Industrial Hygiene Association. For air sampling, field blanks (filters handled on site in the same way as samples but without room air being drawn across them) were analyzed to check for accidental contamination during sample handling and storage. An average of one blank for every ten field samples was analyzed.

Air samples (both static and personal breathing zone) were collected using a validated occupational hygiene method (BV-2017-31103) developed by the Bureau Veritas laboratory. The basis of the method is sampling onto a 25-mm polytetrafluoroethylene (PTFE) filter held in an IOM sampling head followed by solvent extraction and ultrahigh-pressure liquid chromatograph/ultraviolet analysis, with an LLQ of 12 ng per sample and sample stability of at least 28 days under ambient temperature storage conditions. For a sample volume of 30 liters (equivalent to a 15-min sample at 2 liters/min), this analytical limit of quantification is equivalent to a LLQ of 0.4 μg/m^3^.

Part 2 evaluated single and repeat doses of ribavirin-PRINT-IP in participants with moderate COPD. Cohort A (single dose) included 8 participants (6 active, 2 placebo, 60 mg). Cohort B (repeat doses) was planned to include 14 participants (12 active, 2 placebo, 60 mg BID); however, due to the difficulty in recruiting a sufficient number of eligible participants, only a total of 8 participants (6 active, 2 placebo) were enrolled. In the repeat dosing cohort, ribavirin-PRINT-IP was administered for 14 days to mimic the expected duration of treatment in the target patient populations.

The study medication was administered at doses up to 120 mg per day by inhalation using a Monodose RS01 device.

BAL was performed on 14 healthy participants in part 1 (Cohort B) and 8 COPD participants in part 2 (cohort B) to evaluate levels of ribavirin in the ELF. BAL was performed within 1 h after the first morning dose on days 10 to 13.

### Pharmacokinetic assessments.

In both studies, blood samples were collected in ethylenediamine tetra-acetic acid (K_3_EDTA) tubes. Plasma and BAL samples were analyzed for ribavirin using validated analytical methods based on protein precipitation, followed by liquid chromatography and mass spectroscopy analysis (unpublished data). In study 1, the LLQ was 2 ng/ml for assays of both plasma and BAL fluid, using a 50-μl sample aliquot with a higher limit of quantification (HLQ) of 2,000 ng/ml. In study 2, similar methods were used for each sample type, but the LLQ was lowered to 0.25 ng/ml, while the HLQ was reduced to 250 ng/ml.

Blood samples for analysis of urea in plasma were collected into lithium heparin tubes as soon as practically possible before the collection of BAL samples. BAL samples were assayed for urea content and cell counts. BAL samples for epithelial lining fluid analysis were also collected and analyzed for ribavirin concentrations using the assay methods described above.

### Safety assessments.

The safety assessments included the monitoring of AEs, vital signs, pregnancy, and medical device incidents. Physical examinations, clinical laboratory tests, ECGs, spirometry, and capillary partial pressure of carbon dioxide (pCO_2_) were performed during the study.

### Data analysis.

For both study 1 and study 2, no formal statistical hypotheses were tested; all data were descriptively summarized. Plasma ribavirin concentration-time data were analyzed by noncompartmental methods with Phoenix WinNonlin version 6.4 using actual sampling times.

The concentration of ribavirin in the lung was assessed using the concentration of ribavirin in lung ELF from BAL fluid. Urea concentration data from both lung and plasma were used to calculate the dilution effect of the BAL fluid, using the following equation:

$$\begin{array}{c} \text{lung ELF drug concentration}=\text{BAL drug concentration}(\text{ng}/\text{ml})\times \text{dilution factor} \end{array}$$

where

$$\text{dilution factor}=\frac{{\text{Plasma urea}}_{\text{pre-bronch}}}{\text{BAL urea}}.$$
